# *α*TAT1: a potential therapeutic target in cancer?

**DOI:** 10.1038/cddis.2016.46

**Published:** 2016-03-31

**Authors:** J-Y Chien, C-H Lin

**Affiliations:** 1 Institute of Microbiology and Immunology, National Yang-Ming University, Taipei, Taiwan; 2 Institute of Biophotonics, National Yang-Ming University, Taipei, Taiwan; 3 Institute of Clinical Medicine, National Yang-Ming University, Taipei, Taiwan; 4 VYM Genome Research Center, National Yang-Ming University, Taipei, Taiwan

*α*-Tubulin acetyltransferase 1 (*α*TAT1) and its homolog are major *α*-tubulin acetyltransferases conserved in a wide range of species.^1^ In human cells, it catalyzes the reversible acetylation of *α*-tubulin at Lys40. Unlike *α*-tubulin deacetylases, HDAC6 and SirT2, which have multiple substrates, *α*-tubulin is the major substrate of *α*TAT1 identified so far except for its auto-acetylation. These three enzymes largely control the cellular acetylated *α*-tubulin (Ac-Tu) level, which has been suggested to be involved in modulating a variety of cellular functions, including microtubule dynamics, cell migration, and motor protein transport. Despite its role in cellular physiology, depletion of *α*TAT1 did not noticeably affect the viability and growth in several organisms, such as the nematode *Caenorhabditis elegans*, zebra fish, and mice, although morphological defects were found at the tissue or cellular level.^1, 2, 3, 4, 5^

In human cell models, *α*TAT1 depletion was reported to delay ciliogenesis without affecting cell morphology or proliferation in immortalized retinal pigment epithelial cells of RPE-hTERT.^1^ Nevertheless, our recent study published in *Cell Death Discovery*^6^ compared the effect of three *α*TAT1-specific small hairpin RNAs (shRNAs) in human cervix cancer cell line HeLa with lung cancer cell line A549 and revealed that efficient *α*TAT1 downregulation can impair cell proliferation and actin architecture. Using the lentiviral system, we delivered shRNAs to establish stable *α*TAT1-downregulated cells. At about 72 h after delivery, we noticed increasing number of rounded, detached, and abnormally large-sized cells in the more efficient two shRNA-treated groups. Significant decrease in F-actin and focal adhesions were also observed in the most efficient shRNA-treated group.

On further monitoring of cell proliferation by time-lapse microscopy,^6^ we found that detached cells were mainly the cells entering M phase that cannot pass metaphase. HeLa is much more susceptible than A549 in this aspect. Failure at the cytokinesis stage was also increased, usually producing multiploid cells. Over 80% of the control cells entered M phase within the first 36 h of observation, whereas *α*TAT1 downregulation increased the population sustained at interphase in both cell lines (Figure 1a). These characteristics are consistent with mitotic catastrophe,^7^ which could be induced by agents that impair microtubule stability or DNA integrity.

It has been suspected for a long time that acetylation helps to promote the stability of microtubules. After the discovery of Ac-Tu in the green algae *Chlamydomonas* in 1985,^8^ its intracellular distribution has been widely studied using the monoclonal antibody 6-11B-1.^9^ Stable or long-lived microtubules, such as axonemes, and residual microtubules after microtubule-depolymerizing drug treatment are usually enriched with Ac-Tu. During cell division, the kinetochore microtubule is also acetylated shortly after its assembly. However, *in vitro* studies did not support the direct influence of *α*TAT1 or Ac-Tu on microtubule dynamics, suggesting a more complicated relation among them. In our results,^6^ the lack of Ac-Tu did not noticeably affect the shape of mitotic spindle or chromosome alignment at the metaphase in both cell lines. Meanwhile, only marginal change in microtubule outgrowth speed and inter-kinetochore distance was observed. Therefore, it seems worthwhile to consider the possibility that *α*TAT1 also participates in other steps of spindle assembly checkpoint.

The mechanism connecting Ac-Tu and DNA repair was explained in a recent study.^10^ Microtubule-targeting agents that promote microtubule depolymerization, such as vincristine, usually decrease Ac-Tu; on the contrary, agents that promote microtubule polymerization, such as paclitaxel, usually increase Ac-Tu. On monitoring the DNA damage marker *γ*-H2AX, the Ser139 phosphorylated form of histone H2AX accumulated at the DNA double-strand breaks, Poruchynsky *et al.*^10^ found that pretreatment with either vincristine or paclitaxel prolongs the decline of this marker after treatments with DNA-damaging agents. The authors suggested that this sustained γ-H2AX level can underlie the change of microtubule integrity by vincristine or paclitaxel, thereby impairing the transport of DNA repair proteins on it. Our results demonstrated that *α*TAT1 downregulation increased *γ*-H2AX, but not the other two DNA repair response markers, p-CHK1 and p-CHK2.^6^ It is worthy to further differentiate the role of Ac-Tu in the DNA repair response without drastically altering the microtubule integrity.

An intriguing observation that *α*TAT1 depletion-induced deficiencies can be partially rescued by expressing its mutant that cannot acetylate *α*-tubulin,^3^ implying that *α*TAT1 can play other roles independent of its acetylation activity. On the other hand, multiple *α*TAT1 transcription variants are present in cDNA databanks. Most variants consist of a conserved N-terminus, which is sufficient to specifically acetylate *α*-tubulin, and a divergent C-terminus with functions largely unknown. A recent study demonstrated that a mouse *α*TAT1 transcription variant binds *α*-adaptin, which is involved in clathrin-mediated endocytosis, via the C-terminal region, thereby promoting local *α*-tubulin acetylation.^11^ Taken together, we suspect that multiple *α*TAT1 transcription variants are required to maintain different cellular functions through mechanisms partly independent of Ac-Tu (Figure 1b). Therefore, although Ac-Tu level was maintained in HeLa cells stably expressing an *α*TAT1 transcription variant after *α*TAT1 downregulation, this could not be enough to prevent the overall observed deficiencies.^6^

Endogenous Ac-Tu level has recently been linked to metastatic behavior in breast cancer^12^ and *α*TAT1 has been reported to be critical in chemotaxis in the human breast cancer cell line MDA-MB231.^11^ In our study, HeLa and A549 showed different susceptibilities during cell cycle stages after *α*TAT1 downregulation. To date, there is no clear evidence for the impact of mutations at *α*-tubulin Lys40 or *α*TAT1 on humans. Considering that *α*TAT1 depletion did not noticeably affect the viability in mice, differentiating the role of *α*TAT1 in more types of cancer and its potential as a therapeutic target are worthy of further investigation.

## Figures and Tables

**Figure 1:**
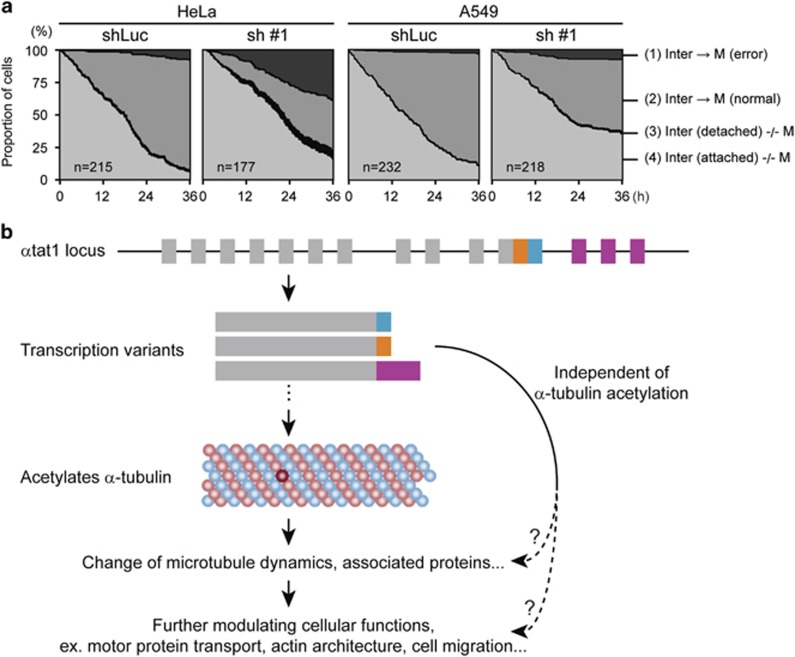
(**a**) Efficient *α*TAT1 downregulation increases M-phase error and cells sustain at interphase in HeLa and A549 cells. Cell fate until the first round M-phase completion in time-lapse recordings was traced manually and divided into four groups: (1) entered M phase and error at metaphase or cytokinesis was observed, (2) entered M phase and produced two daughter cells, (3) detached before entering M phase, and (4) attached but not yet entering M phase. Time of the anaphase onset represents M-phase time point. This figure is also used in our work published in *Cell Death Discovery*^6^ (doi:10.1038/cddiscovery.2016.6). (**b**) Suspected model of multiple *α*TAT1 transcription variants were required to maintain different cellular functions through mechanisms partly independent of Ac-Tu
